# Collectanea Medica: Consisting of Anecdotes, Facts, Extracts, Illustrations, &c.

**Published:** 1820-05

**Authors:** 


### COLLECTANEA MEDICA:

CONSISTING OF

### ANECDOTES, FACTS, EXTRACTS, ILLUSTRATIONS, &c.

***Relating to the History or the Art of Medicine, and the Auxiliary Sciences.***

Floriferis ut apes in saltibus omnia libant,
Omnia nos itidem depascimur aurea dicta.

### *Observations respecting the Gymnotes, and other Electric Fish.*

[From Baron HUMBOLDT's *Historical Relation of his Voyage in America.*—In continuation from page 302.]

A PERSON cannot expose himself with impunity to the first shocks of a large gymnotus when much irritated. If, by chance, we receive a stroke before the fish is wounded or fatigued by a long pursuit, the pain and subsequent numbness are so intense, that it is impossible to describe the sensations we experience. I do not recollect to have ever received, by the discharge of a large Leyden jar, a more terrible shock than that which I have experienced on imprudently placing both my feet on a gymnotus which had just been taken from the water. I was affected during the rest of the day with acute pain in the knees, and in almost all the joints. In order to discern the remarkable difference there is between the sensation produced by the voltaic pile and electric fish, it is necessary for us to touch the latter when they are in a state of extreme weakness: gymnotes and torpedoes cause then a subsultus tendinum, which is propagated from the finger to the elbow. We seem to experience, at each stroke, an internal vibration which continues for two or three seconds, and which is followed by a painful sort of numbness. The sensations caused by a weak gymnotus have appeared to me to be very similar to the painful tremulation with which I have been seized on each contact of two heterogeneous metals, applied to wounds made by means of cantharides. This difference between the sensations produced by electric fish and the pile, or a Leyden jar weakly charged, has struck all observers: it does not, however, oppose the idea of the identity of electricity and the galvanic agency of fishes. The electricity may be the same; but its effects may be differently modified by the disposition of the electric apparatus, by the intensity of the fluid, by the rapidity of the stream, and by a peculiar mode of agency.

In Dutch Guyana,—at Demarara, for example, gymnotes were formerly employed for the cure of paralytic affections. These cures by electricity are also heard of amongst the savages of America; and we find evidences of them amongst the Greeks. Scribonius Largus, Galen, and Dioscorides, speak of torpedoes having cured affections of the head and gout. I have not heard any thing of this remedy in the Spanish colonies I have passed through; but I can assert, that, after having made experiments with gymnotes for four hours in succession, we suffered (Bonpland and myself), debility of the muscles, pain in the joints, and general uneasiness, which continued until the next day, and which were the effects of great irritation of the nervous system.

Gymnotes are neither charged conductors, nor batteries, nor electric apparatuses, from which shocks are received every time they are touched by the hand, or on applying both hands so as to form a conductive circle between heterogeneous poles. The electric action of the fish depends solely on its will. We may often touch the animal, isolated and non-isolated, without experiencing any shock. When M. Bonpland held it by the head or the middle of the body, whilst I held it by the tail, and, standing on moist ground, we joined the other hands, one of us received shocks that the other did not experience. It seems to be in the power of the gymnotus to act only at the point where he is most strongly irritated. The discharge then takes place only at one point, and not at the neighbouring parts. When two persons touch with a finger the belly of the animal, at an inch distance from each other, and press it simultaneously, it is sometimes one and sometimes the other that receives the shock. So, when a person isolated holds the tail of a vigorous gymnotus, and another person pinches it about the branchiæ and the pectoral fin, it is often only the first who suffers the shock. It did not appear to us that this difference could be attributed to the dryness or humidity of our hands, or their unequal conductibility. The gymnotus seemed to direct his strokes sometimes throughout the whole surface of its body, and sometimes through only a certain part. This effect does not so much indicate a partial discharge of the organ composed of an innumerable quantity of filaments, as a faculty possessed by the animal (perhaps by the instantaneous secretion of a fluid which is evolved in the cellular tissue), to establish a communication of its organs with the skin only in a very limited extent.

Nothing better proves the faculty possessed by the gymnotus (by the influence of its brain and nerves), to dart and direct its stroke at will, than the observations made at Philadelphia, and recently at Stockholm,* on gymnotes rendered extremely tame. When they had fasted for a long time, they killed small fish, put into a vessel of water with them, at a great distance; that is to say, their electric shock traversed a very thick medium of water. We must not be surprised that they have observed in Sweden, in a single gymnotus, what we were not able to see in a great number in their native country. As the electric action of the animals is a *vital action*, and under the dominion of their will, it does not depend solely on their health or vigour. A gymnotus carried from Surinam to Philadelphia and to Stockholm, had become accustomed to his prison, and he had gradually acquired, in the vessel in which he was kept, the same habits as he had in his native rivers and pools.

They brought to us, at Calabozo, an electric eel taken in a net, consequently, without having been wounded. It ate food very well, and terribly frightened some little tortoises and frogs, which, ignorant of the danger, placed themselves with confidence on the back of the fish. The frogs did not receive the shock until the instant at which they touched the body of the gymnotus. Having recovered from it, they saved themselves by leaping out of the pail; and, when they were replaced near the fish, the mere sight of it terrified them. We did not then observe anything which indicated an action at a distance; but this gymnotus, recently taken, was not sufficiently tamed to permit him to attack and devour frogs. On approaching a finger, or metallic rods, to the distance of half a line from the electric organs, no shock was felt. The animal did not, perhaps, perceive the near approach of the foreign body; or, if he did perceive it, it may be supposed, that the timidity he preserved at an early period of his captivity led him to dart his shocks only when irritated by immediate contact. The gymnotus being plunged in water, I approached my hand, both with and without metal, to within the distance of a few lines from the electric organs, without suffering any shock; whilst M. Bonpland

---

* By WILLIAMSON and FAHLBERG. The following are the most interesting observations published by the latter, (in the *Vetensk. Acad. my. handl. quart.* 2, 1801:) "The gymnotus sent from Surinam to Stockholm, to Mr. AORDERLING, lived more than four months in a state of perfect health. It was twenty-seven inches in length, and the shocks it gave were so violent, especially when in the air, that I could find hardly any means for preserving myself from them by non-conductors, when I moved the fish from one place to another. Its stomach was very small: it ate but very little at a time, but often. It approached living fish, and darted at them, from a distance, a shock, the energy of which was proportionate to the size of the prey. It was but rarely deceived in its judgment: a single stroke was almost always sufficient to overcome the resistance presented by the medium of water, which the electric stream had to traverse. When it was much urged by hunger, he would also dart strokes at the man who daily fed him. It lost almost entirely its electric power a short time before its death."

strongly irritated the animal by immediate contact, and received from it very violent strokes.

The electric organ of the gymnotus acts only under the immediate influence of the brain and the heart. After dividing a very vigorous fish through the middle of the body, I found that only the external part would give me shocks. The strokes are equally powerful in whatever part of the body the fish is touched; but it is most disposed to dart them, when the pectoral fin, the electric organ, the lips, the eyes, and the branchiæ, are touched. Sometimes the animal struggles forcibly with a person who holds him by the tail, without communicating the least shock to him: neither did I experience any, when I made a slight incision near its pectoral fin, and galvanised the wound by the simple contact of two plates of zinc and silver. The gymnotus curled itself in a convulsive manner; it raised its head out of the water, as if alarmed by a sensation so new to it. The most violent muscular motions are not always accompanied with electric discharges.

The action of the fish on the organs of man, is transmitted and intercepted by the same bodies which transmit and intercept the electric stream of a conductor charged by the Leyden jar or the voltaic pile. In wounded gymnotes, which cause but slight though very equal shocks, these shocks have appeared to me to be constantly more violent when the fish was touched by the hand armed with metal, than by the hand alone. They are also more intense, when, instead of touching it by one armed or unarmed hand, both the hands, in either of those states, are applied together to the animal. When two persons, isolated or non-isolated, hold each other by the hand, and only one of them touches the fish with the other, either alone or armed with metal, shocks are commonly experienced by both persons at the same time. It however also happens sometimes, that, in the most painful shocks, only the person who immediately touches the fish experiences them. When the gymnotus, being exhausted, or in a state of very feeble excitability, will not dart any more strokes on being irritated by a single hand, shocks are very severely felt on forming a chain and employing both hands. However, even in this case, the electric shock takes place only at the will of the animal. Two persons, one of whom holds its tail and the other its head, cannot force the gymnotus to dart a stroke when they take each other by the hand, and thus form a chain. On employing, in many different ways, very susceptible electrometers, isolating them on a plate of glass, and receiving very violent strokes, which passed through the electrometer, I have never been able to discover any phenomena of attraction or repulsion. The same remark has been made, at Stockholm, by Mr. Fahlberg. This naturalist, however, has seen an electric spark, as Walsh and Ingenhousz had done before him, on placing the gymnotus in the air, and on interrupting the conductive chain by two plates of gold stuck on glass, and separated to the distance of a line. But no person, on the contrary, has ever perceived a spark coming from the body of the fish. We irritated one for a long time in the night, at Calabozo, in perfect darkness, but we observed no luminous phenomenon. On disposing four gymnotes of unequal strength, in such a manner that I received the shocks from the most vigorous fish *by communication*, that is to say, only by touching one of the other fish, I could not perceive any agitation in the latter at the instant when the electric stream passed through their body. Perhaps the stream passed merely by the medium of the humid surface of their skin. We should not, however, thence conclude, that gymnotes are insensible to electricity. It is fair to consider, that their nervous system is affected by the same agents as those of other animals. I have indeed seen, that, on laying bare their nerves, they suffered muscular contractions on the simple contact of two heterogeneous metals; and Mr. Fahlberg found that his gymnotus was convulsed when it was placed in a copper vessel, and slight discharges from a Leyden jar passed through its skin.

After the experiments I had made on gymnotes, I felt a great desire, on my return to Europe, to ascertain precisely the different circumstances in which another electric fish, the torpedo of our seas, will give electric shocks. Although this fish has been examined by a great number of naturalists, what has been published on its electric effects is extremely vague. It has been gratuitously supposed that it acts like a Leyden jar, which is discharged at will, on being touched by the hands, and this supposition appears to have led those who have engaged in researches of this kind into many errors. M. Gay-Lussac and me, during our voyage in Italy, made a great many experiments on torpedoes taken in the gulf of Naples. These experiments presented several results very different from those I had witnessed from those on the gymnotus. It is probable, that these variations depend rather on an inequality of the electric power of the two fishes, than on a different disposition of their organs.*

Although the force of the torpedo cannot be compared to that of the gymnotus, it is sufficient to produce very painful sensations. A person accustomed to electric shocks holds with difficulty in his hands a vigorous torpedo of twelve or fourteen inches in length. When the animal gives no longer any more than very feeble strokes when plunged in water, the shocks become very sensible if it be raised above the surface of this fluid. I have often observed this phenomenon on galvanising frogs. The torpedo agitates in a convulsive manner its pectoral fins every time it darts a stroke, and the shock from it is more or less painful, according as the immediate contact takes place by a more or less extensive surface. I have observed above, that the gymnotus produces the most violent shocks without exerting any sensible motions of the eyes, the head, or the pectoral fins.† Does this difference depend on the position of the electric organ, which is not double in the gymnotus? or does the motion of the pectoral fins of the torpedo directly prove that this fish re-establishes the electric equilibrium by its own skin; that it discharges itself by its own body; and that we only experience generally the effect of a lateral shock?

Neither the torpedo nor the gymnotus can be discharged at will, as

---

* Geoffroy Saint-Hilaire, *Annales du Museum*, tom. i. p. 392—407.

† The anal fin alone is sensibly agitated in the gymnotus when this fish is excited under the belly, where the electric organ is situate.

we can discharge the Leyden jar or the voltaic pile. A shock is not always experienced, even when an electric fish is touched with both hands; it is necessary to irritate it, to cause it to produce a stroke. It is quite indifferent whether or not the person who touches it be isolated. What has been said of the necessity of a communication with moist ground to establish a chain, is founded on incorrect observations.

M. Gay-Lussac has made the important observation, that, when an isolated person touches the torpedo with only one finger, it is absolutely necessary that the contact be immediate, in order that a shock may be received. The fish is touched with impunity with any metallic instrument; no shock is felt when a conductor or non-conductor is interposed between the finger and the electric organ of the animal. This circumstance shows a great difference between the torpedo and the gymnotus; the latter passing his strokes through a bar of iron several feet in length.

When a torpedo is placed on a metallic plate of but little thickness, so that the plate touches the part of the animal corresponding to the electric organs, the hand which sustains the plate experiences no commotion, although another person, isolated, irritates the animal, and the convulsive motion of its pectoral fins announces strong and reiterated discharges. If, on the contrary, a person sustains with the left hand a torpedo placed on a metallic plate, as in the foregoing experiment, and then touches the upper surface of the electric organ with his right hand, a powerful shock is then felt in both arms. The same sensation is experienced when the fish is put between two metallic plates, not in contact in any part, and the hands then applied to the outer surfaces of the plates. Thus, the interposition of a single plate prevents the shock when only one hand is applied, whilst the interposition of two plates does not prevent it when both hands are employed. In the latter case, it cannot be doubted but that the circulation of the fluid is established by both arms. If, the fish being in the same position between the two plates, there exists some immediate communication between the edges of the two plates, all commotion ceases. The chain between the two surfaces of the electric organ is then formed by the plates, and the new communication which is established by the contact of the two hands with the plates exists without effect. We have with impunity carried the torpedo between two plates of metal, not experiencing the strokes he darted, except when the two plates of metal did not come in contact at some part of their margins.

In the torpedo, as in the gymnotus, nothing seems to show that the animal modifies the electricity of the bodies which surround him. The nicest electrometer is not at all affected, in whatever way it be employed, either on approaching it towards the electric organ of the fish, or on isolating the animal, or on covering it with a metallic plate, and making this plate communicate by a conducting wire with the voltaic condensor. We have varied these experiments with great care; they have always shown the same negative results, and fully confirm what M. Bonpland and myself observed on the gymnotes in South America.

Electric fish, when they are very vigorous, act with equal energy under water and in the air. This observation led us to examine the conductive power of water; and we have found that, when several persons form a chain between the superior and inferior surface of the electric organ of the torpedo, the shock is felt only when those persons have wet hands. The action is not intercepted, if two persons, who sustain the torpedo with their right hands, instead of taking each other by the left hand, each dip a metallic rod in a drop of water placed on an isolating body. On substituting flame for the drop of water, the communication is intercepted, and is not re-established, as in the gymnotes, until the two rods immediately touch each other in the middle of the flame.

Dr. Schilling stated that the gymnotus approached the magnet apparently in an involuntary manner. We were astonished to find this idea adopted by Pozo. We have tried in a thousand ways to discover this pretended influence of the magnet on the electric organs, and we have never observed any sensible effect. The fish did not approach a magnet any more than it did a bar of iron not magnetized. Iron filings thrown on its back showed no sensible motion.

The gymnotes, subjects of so much interest to European naturalists, are dreaded and detested by the native inhabitants of South America. They offer, it is true, good food, in their muscular structure; but the electric organ occupies the greatest part of their body, and this organ is glairy, and disagreeable to the taste: they therefore carefully separate it from the rest of the body. They consider the presence of the gymnotes as the chief cause of the want of other fish in the ponds and pools of the Llanos. They kill more fish than they eat; and the Indians assured us, that, when young crocodiles were taken in strong nets with gymnotes, the latter never showed any signs of wounds, because they benumbed the crocodiles before they could be attacked by them. All the inhabitants of the water dread the society of the gymnotes. Lizards, tortoises, and frogs, seek pools where they may live secure from their influence. It has been necessary to alter the direction of a road near Writuen, because the electric eels have so accumulated in a river there, that they killed, every year, a great number of loaded mules which forded it at that place.

